# Supplementary material for: The Chimeric Peptide (GEP44) Reduces Body Weight and Both Energy Intake and Energy Expenditure in Diet-Induced Obese Rats
Source: bioRxiv. 2025 Feb 22:2025.01.06.631534. Originally published 2025 Jan 7. Preprint. [Version 3] doi: 10.1101/2025.01.06.631534 (PMC11741413; doi:10.1101/2025.01.06.631534)
Supplement: Supplement 1 — Supplemental Table 1. Range of detectability for plasma hormones. Supplemental Figure 1: Schematic of Experimental Paradigms Used in Studies 1, 2 and 3. Supplemental Figure 2A-B: Effects of the chimeric peptide, GEP44, or selective GLP-1R agonist, exendin-4, on core temperature in male and female HFD-fed rats. Male (N=15/group) and female (N=7/group) rats were maintained on HFD (60% kcal from fat for at least 4 months prior to being implanted with PDT-4000 telemetry devices into the abdominal cavity. During this study, animals remained in their home cages and subsequently received SC injections of vehicle (sterile saline/water) followed by GEP44 (50 nmol/kg; 1 mL/kg injection volume) or exendin-4 within 15 min prior to the start of the dark cycle in a counterbalanced design. Core temperature data was collected using the PDT-4000 telemetry devices. A, Effect of GEP44 on core temperature during the light and dark cycle periods in A) male and female HFD-fed rats; B, Effect of exendin-4 on core temperature during the light and dark cycle periods in B) male and female HFD-fed rats. Core temperature was averaged at 11-h light and 12-h dark cycle periods over the 2-day vehicle and 2-day drug treatment period. Data are expressed as mean ± SEM. *P<0.05 GEP44 or exendin-4 vs. vehicle. Supplemental Figure 3A-D: Effects of the chimeric peptide, GEP44, or selective GLP-1R agonist, exendin-4, on gross motor activity over 6-h post-injection in male HFD-fed rats. Male (N=15/group) rats were maintained on HFD (60% kcal from fat for at least 4 months prior to being implanted with PDT-4000 telemetry devices into the abdominal cavity. During this study, animals remained in their home cages and subsequently received SC injections of vehicle (sterile saline/water) followed by GEP44 (50 nmol/kg; 1 mL/kg injection volume) or exendin-4 within 15 min prior to the start of the dark cycle in a counterbalanced design. Gross motor activity data was collected using the PDT-4000 telemetry devices. [file NIHPP2025.01.06.631534v3-supplement-1.pdf]

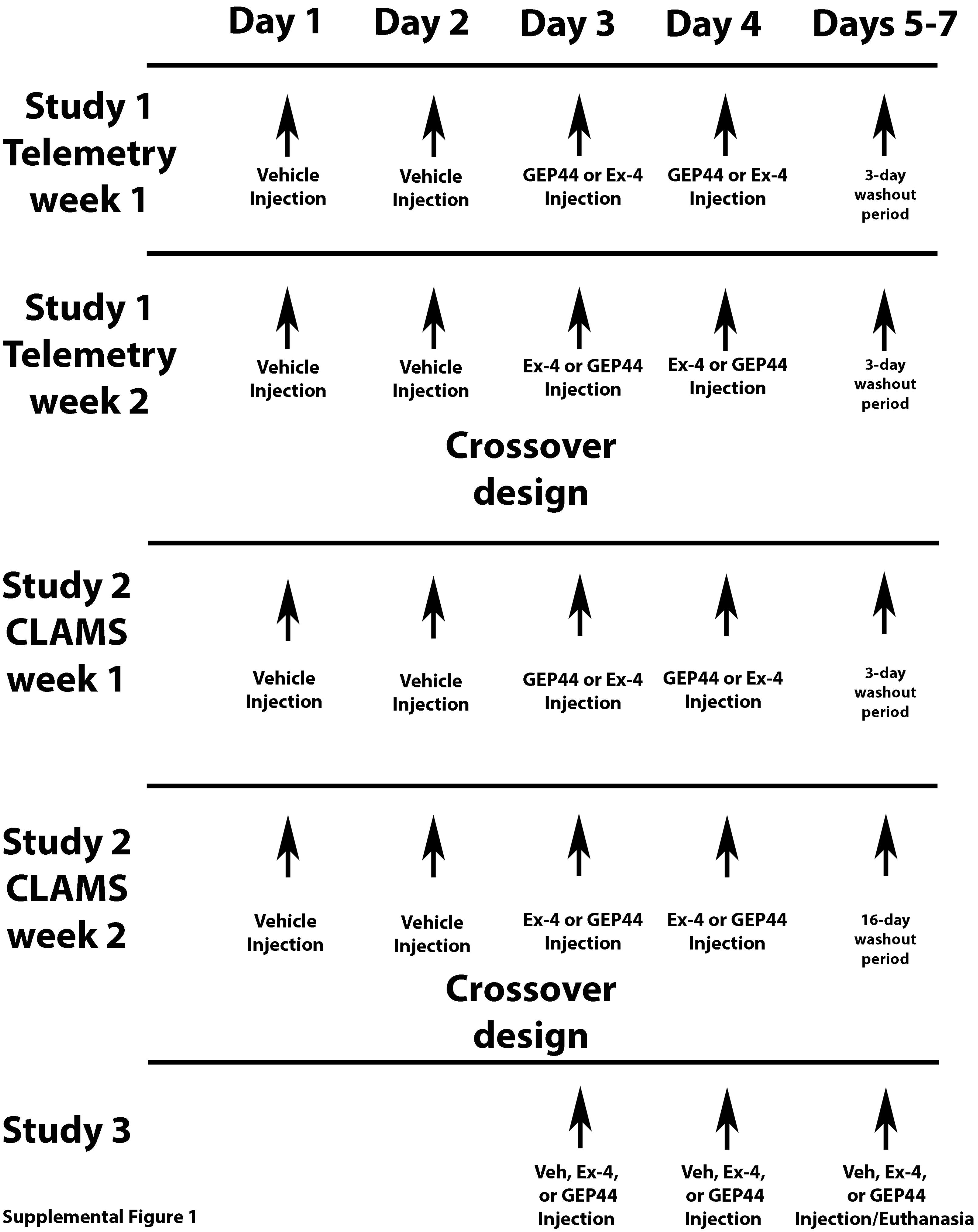

Supplemental Figure 1

Effects of GEP44 and Exendin-4 on Core Temperature in Male and Female HFD-Fed Rats

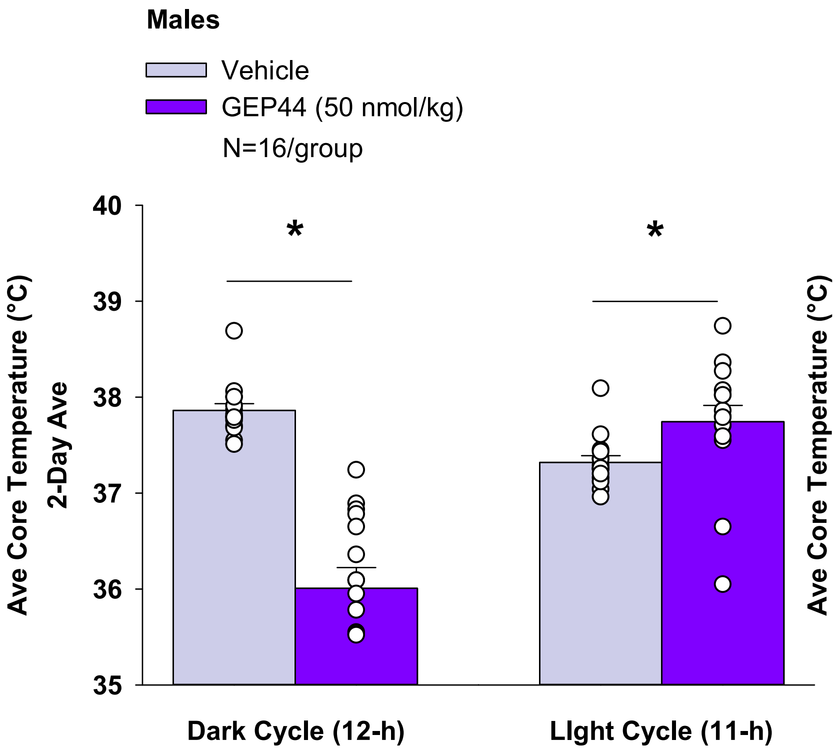

Supplemental Figure 2A

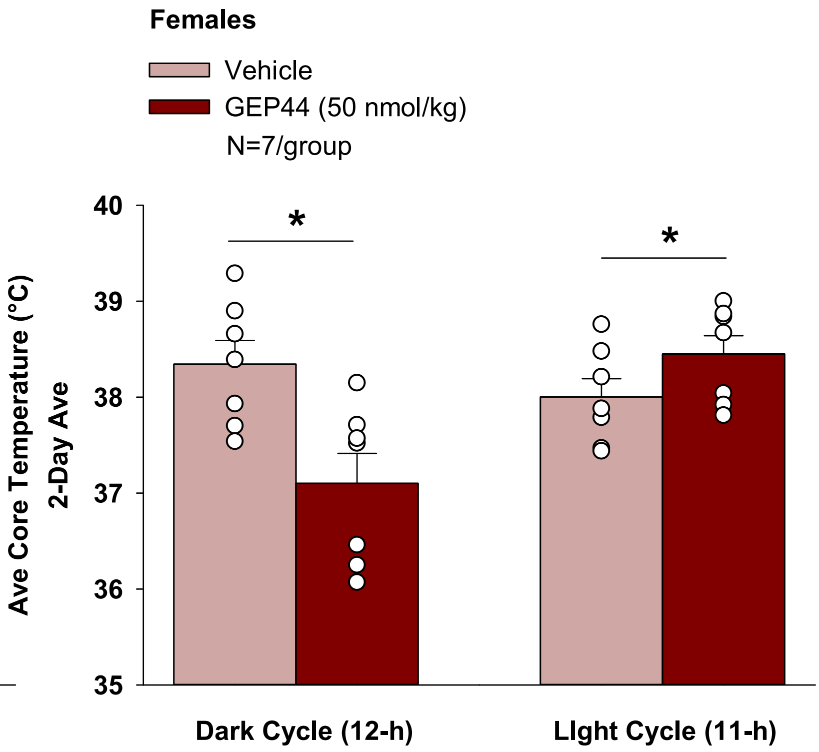

Supplemental Figure 2B

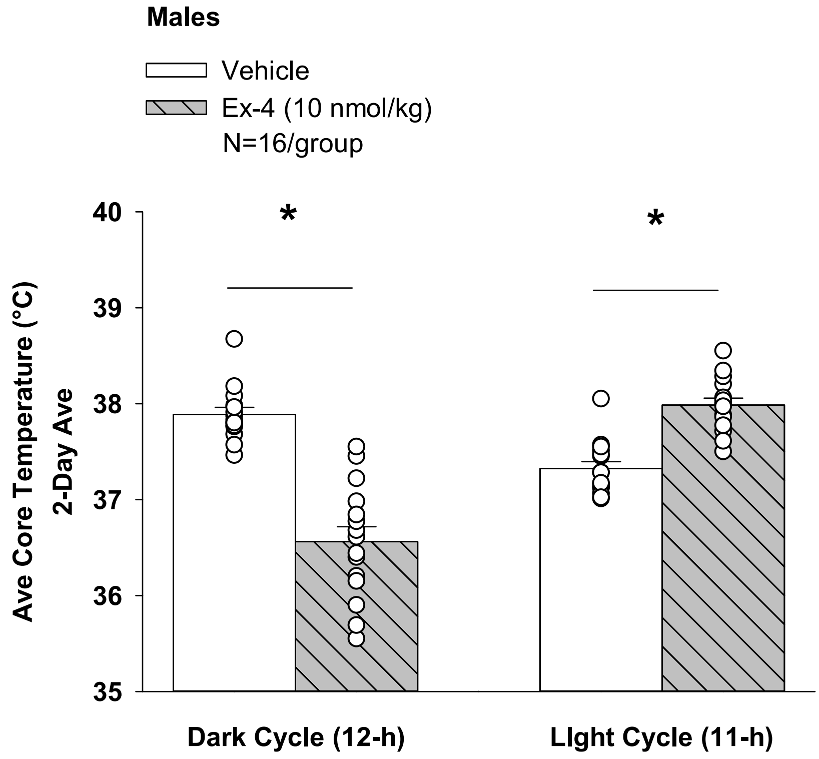

Supplemental Figure 2C

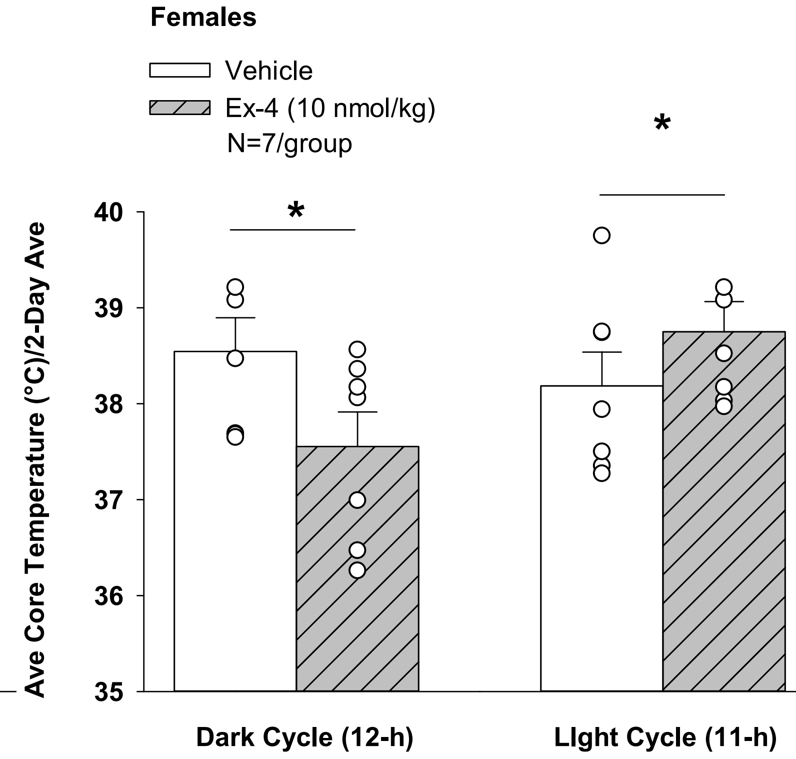

Supplemental Figure 2D

Effects of GEP44 and Exendin-4 on Activity in Male HFD-Fed Rats

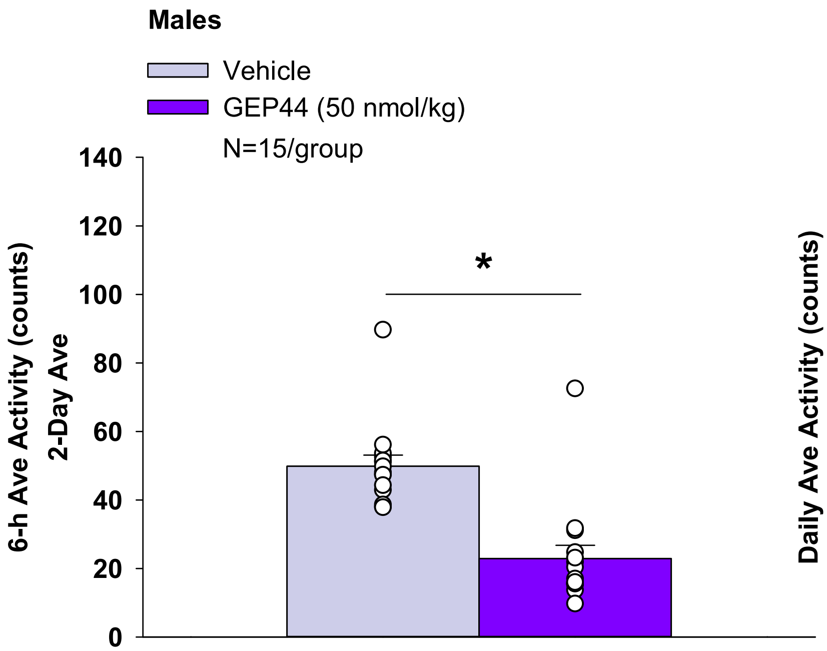

Supplemental Figure 3A

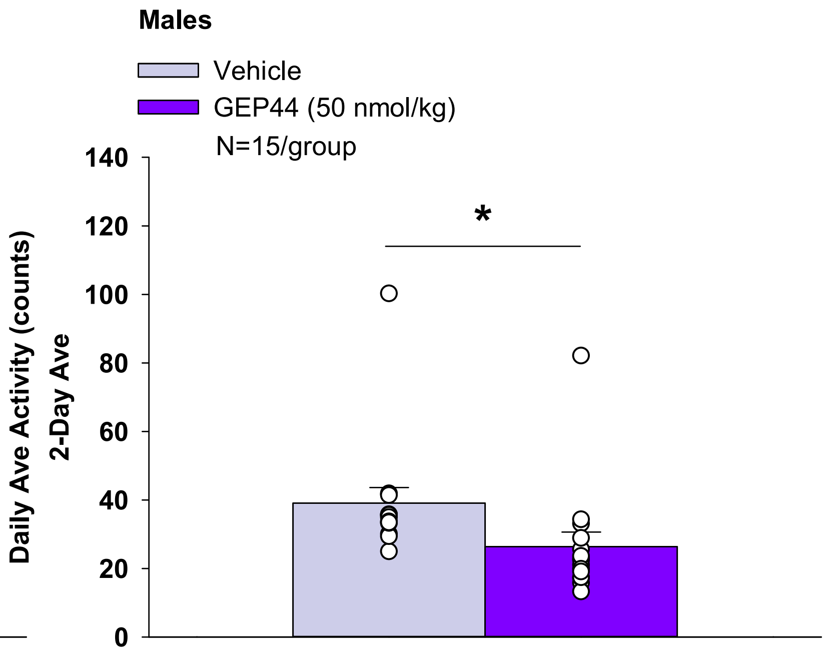

Supplemental Figure 3B

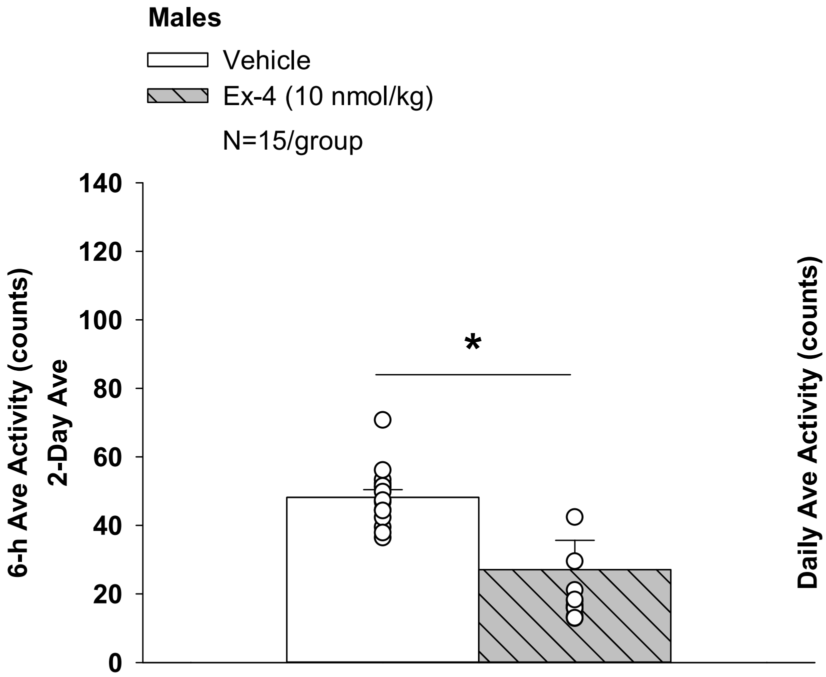

Supplemental Figure 3C

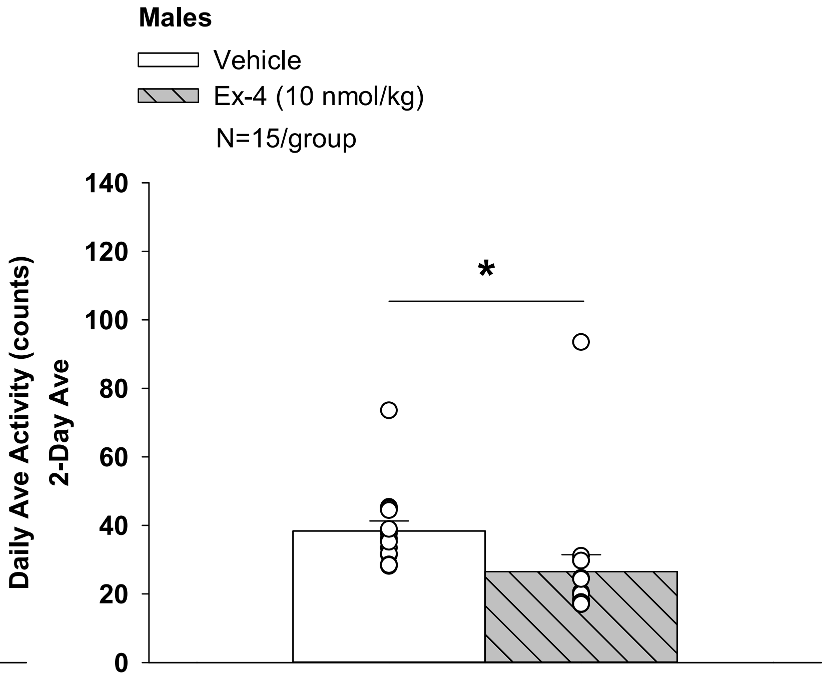

Supplemental Figure 3D

Effects of GEP44 and Exendin-4 on Activity in Male HFD-Fed Rats

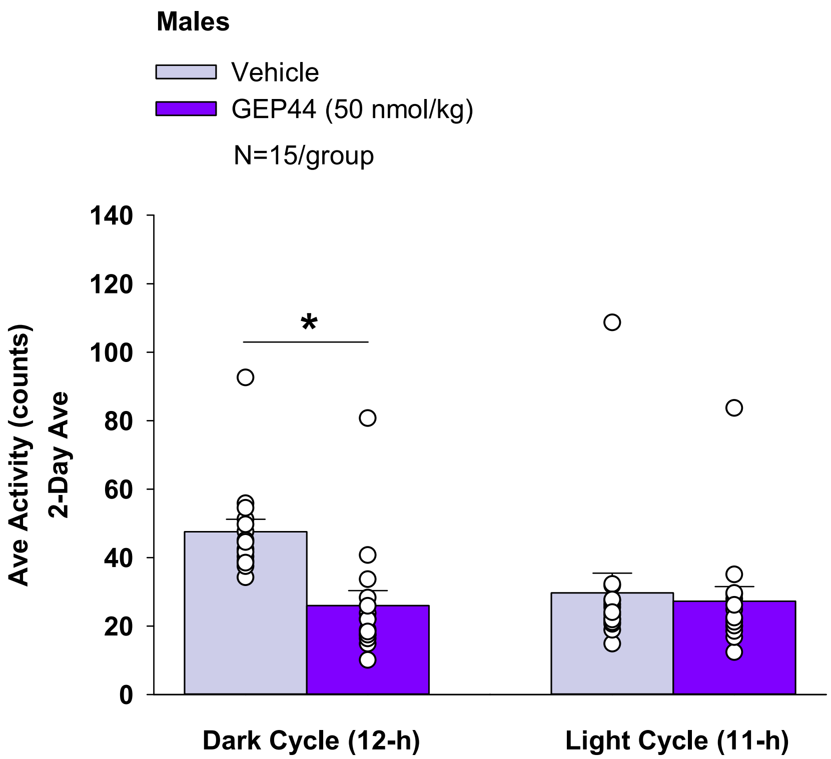

Supplemental Figure 4A

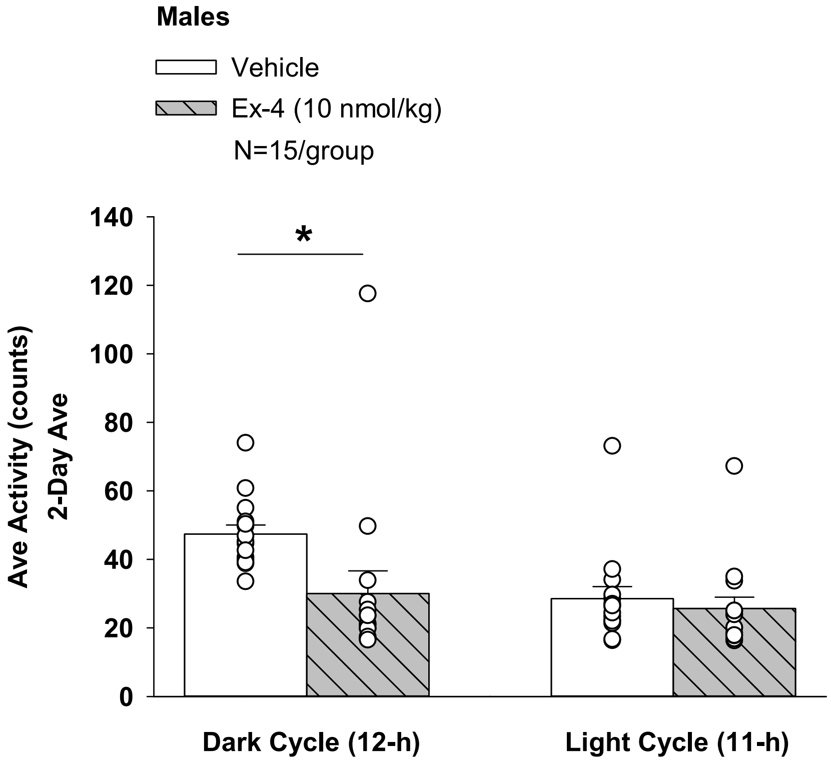

Supplemental Figure 4B

bioRxiv preprint doi: https://doi.org/10.1101/2025.01.06.631534; this version posted February 22, 2025. The copyright holder for this preprint (which was not certified by peer review) is the author/funder. This article is a US Government work. It is not subject to copyright under 17 USC 105 and is also made available for use under a CC0 license.

Supplemental Table 1. Range of detectability for plasma hormones

Plasma hormone	Range of Detectability
Adiponectin	0.25-10 ng/mL
FGF-21	0.0313-2 ng/mL
Glucagon	6.97-633.9 pg/mL
Insulin	0.069-50 ng/mL
Irisin	78-5,000 ng/mL
Leptin	0.07-51.9 ng/mL
