# Supplementary material for: The Chimeric Peptide (GEP44) Reduces Body Weight and Both Energy Intake, and Both Energy Expenditure in Diet-Induced Obese Rats
Source: bioRxiv. 2025 Feb 2:2025.01.06.631534. Originally published 2025 Jan 7. Preprint. [Version 2] doi: 10.1101/2025.01.06.631534 (PMC11741413; doi:10.1101/2025.01.06.631534)
Supplement: Supplementary file 1 [file NIHPP2025.01.06.631534v2-supplement-1.pdf]

bioRxiv preprint doi: https://doi.org/10.1101/2025.01.06.631534; this version posted February 3, 2025. The copyright holder for this preprint (which was not certified by peer review) is the author/funder. This article is a US Government work. It is not subject to copyright under 17 USC 105 and is also made available for use under a CC0 license.

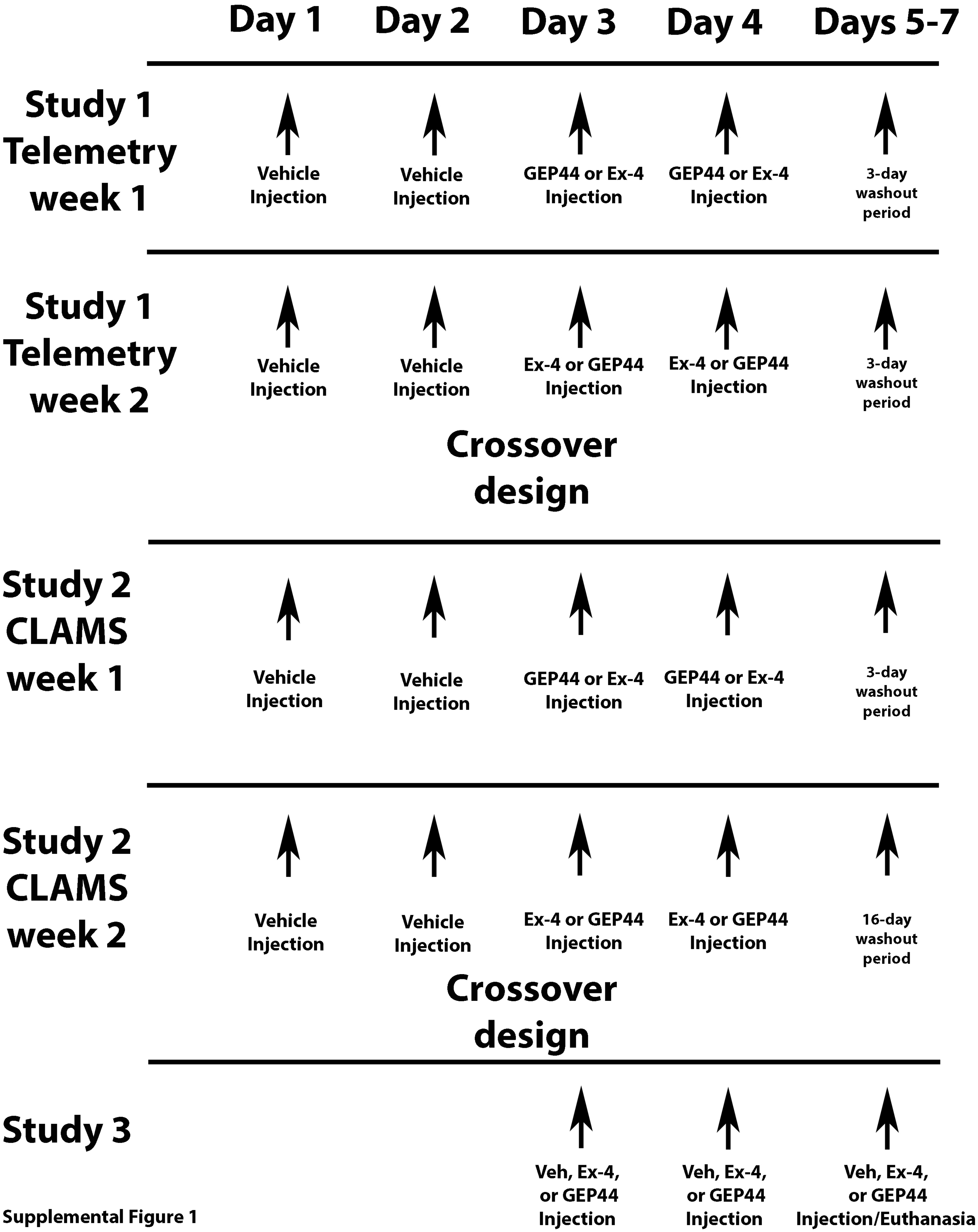

Supplemental Figure 1

bioRxiv preprint doi: https://doi.org/10.1101/2025.01.06.631534; this version posted February 3, 2025. The copyright holder for this preprint (which was not certified by peer review) is the author/funder. This article is a US Government work. It is not subject to copyright under 17 USC 105 and is also made available for use under a CC0 license.

Effects of GEP44 and Exendin-4 on Core Temperature in Male and Female HFD-Fed Rats

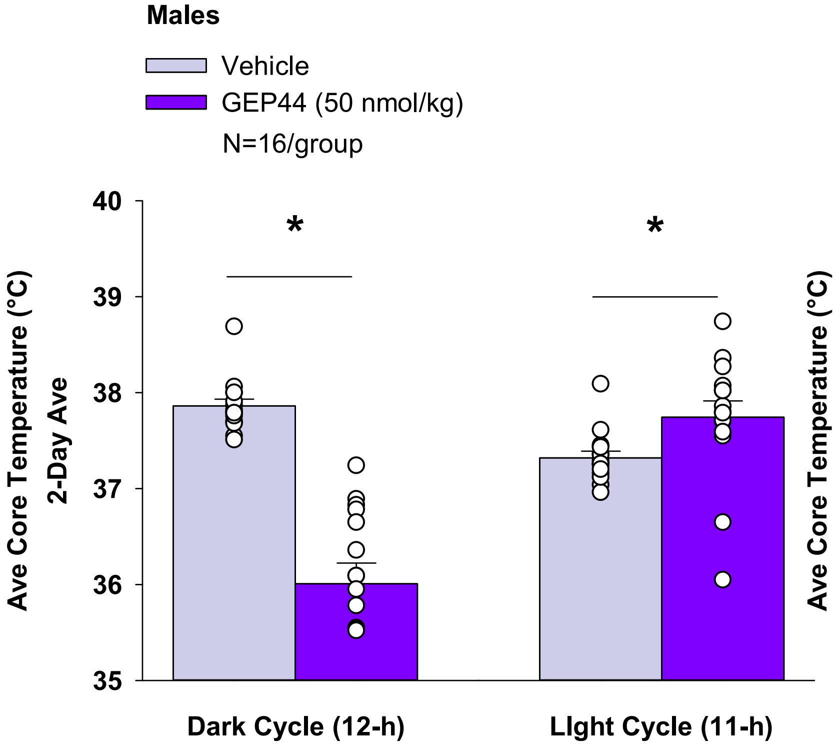

Supplemental Figure 2A

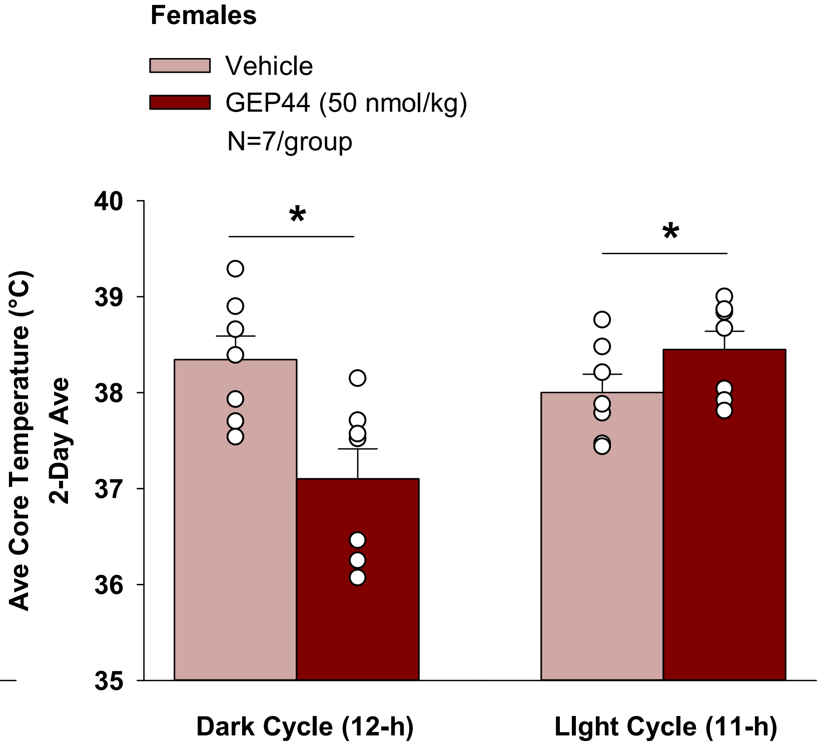

Supplemental Figure 2B

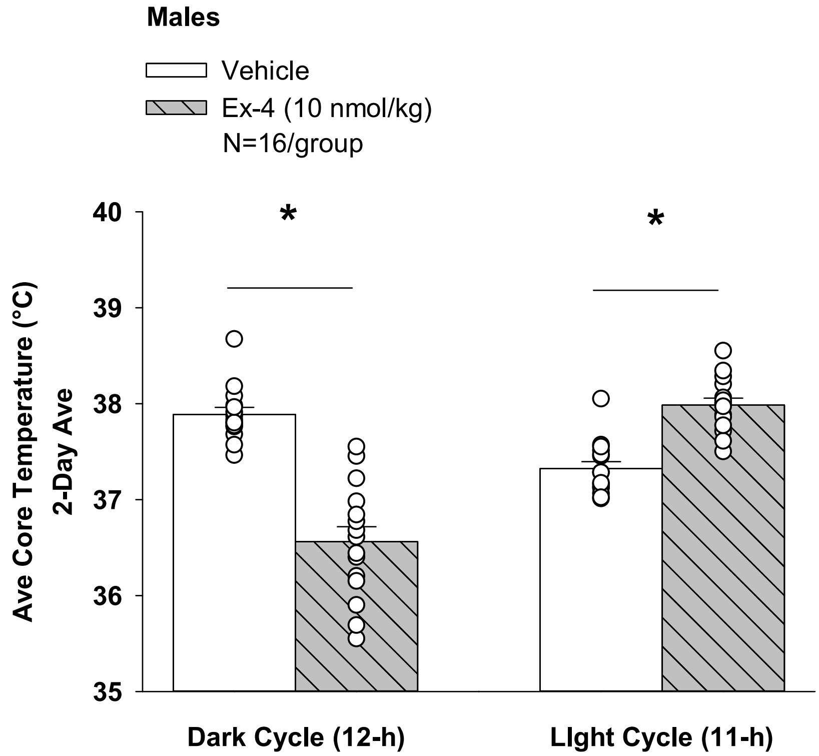

Supplemental Figure 2C

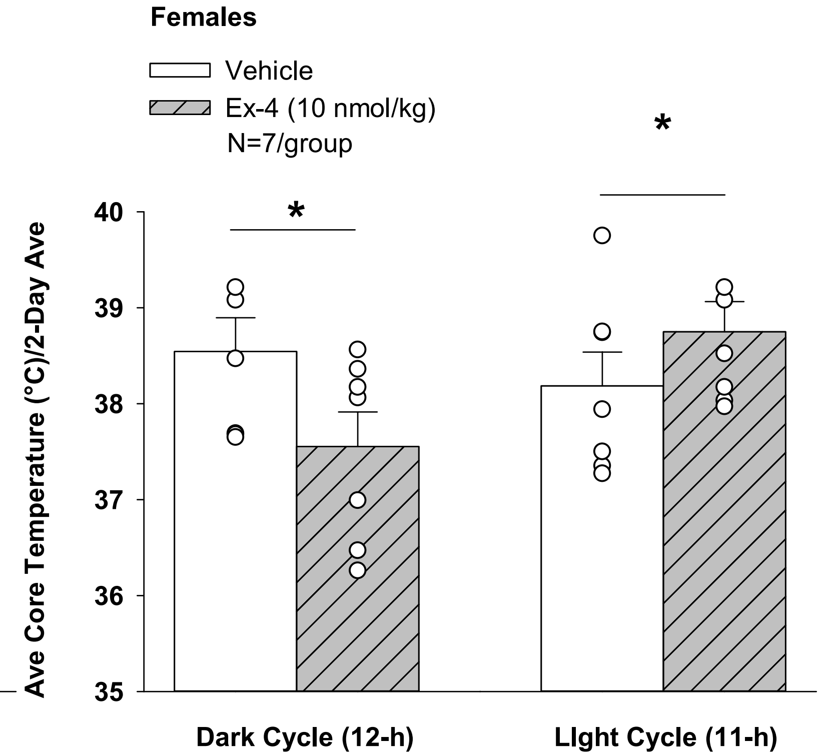

Supplemental Figure 2D

bioRxiv preprint doi: https://doi.org/10.1101/2025.01.06.631534; this version posted February 3, 2025. The copyright holder for this preprint (which was not certified by peer review) is the author/funder. This article is a US Government work. It is not subject to copyright under 17 USC 105 and is also made available for use under a CC0 license.

Effects of GEP44 and Exendin-4 on Activity in Male HFD-Fed Rats

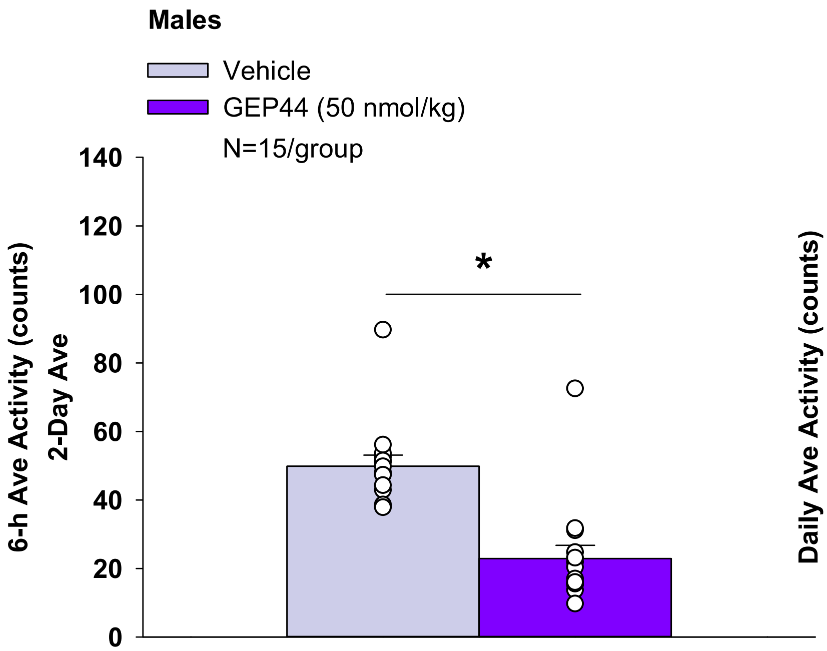

Supplemental Figure 3A

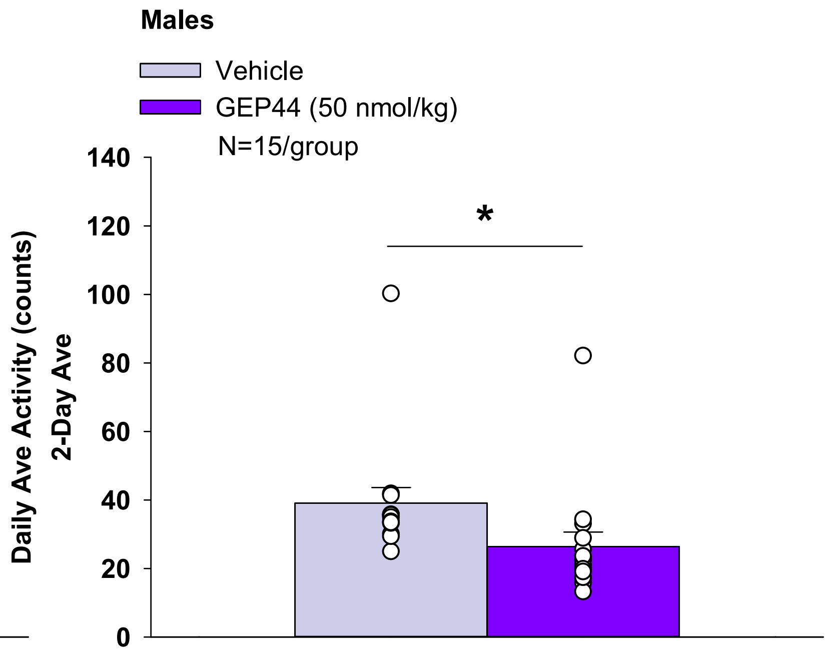

Supplemental Figure 3B

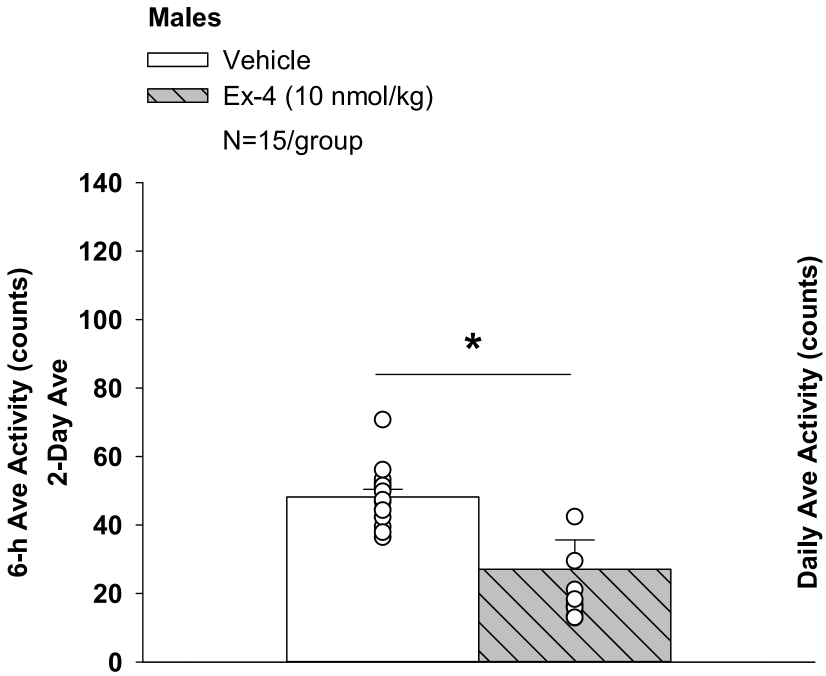

Supplemental Figure 3C

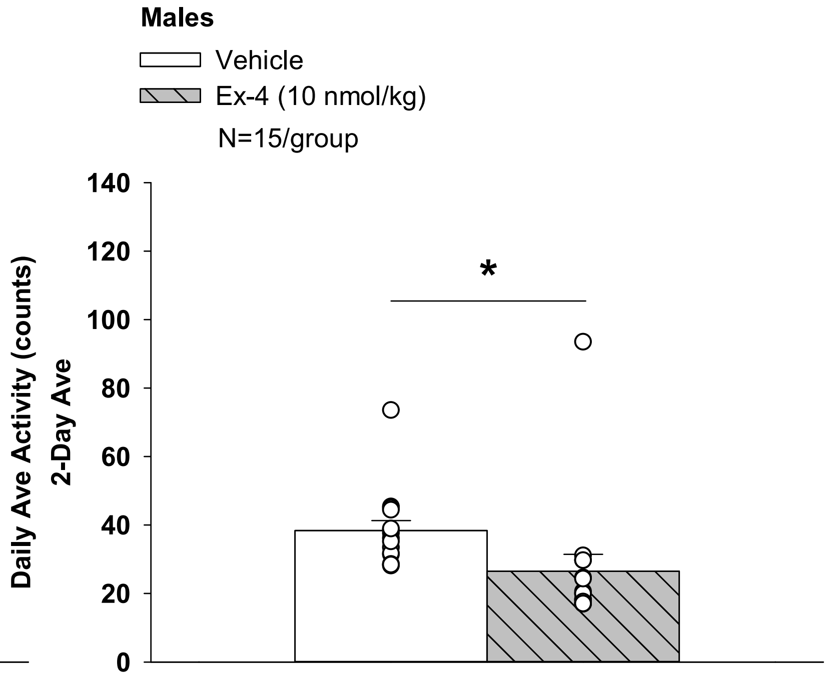

Supplemental Figure 3D

bioRxiv preprint doi: https://doi.org/10.1101/2025.01.06.631534; this version posted February 3, 2025. The copyright holder for this preprint (which was not certified by peer review) is the author/funder. This article is a US Government work. It is not subject to copyright under 17 USC 105 and is also made available for use under a CC0 license.

Effects of GEP44 and Exendin-4 on Activity in Male HFD-Fed Rats

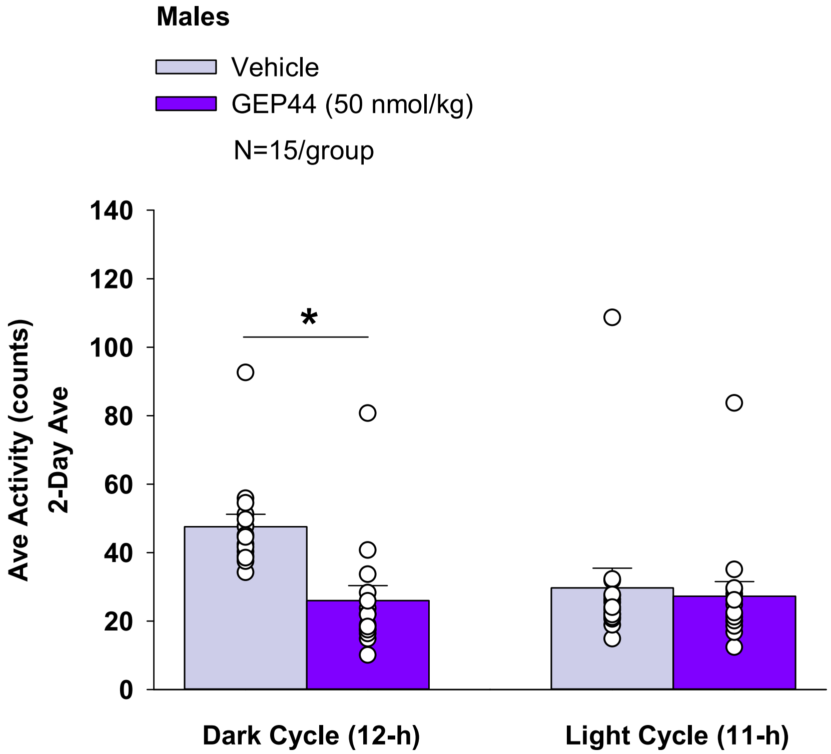

Supplemental Figure 4A

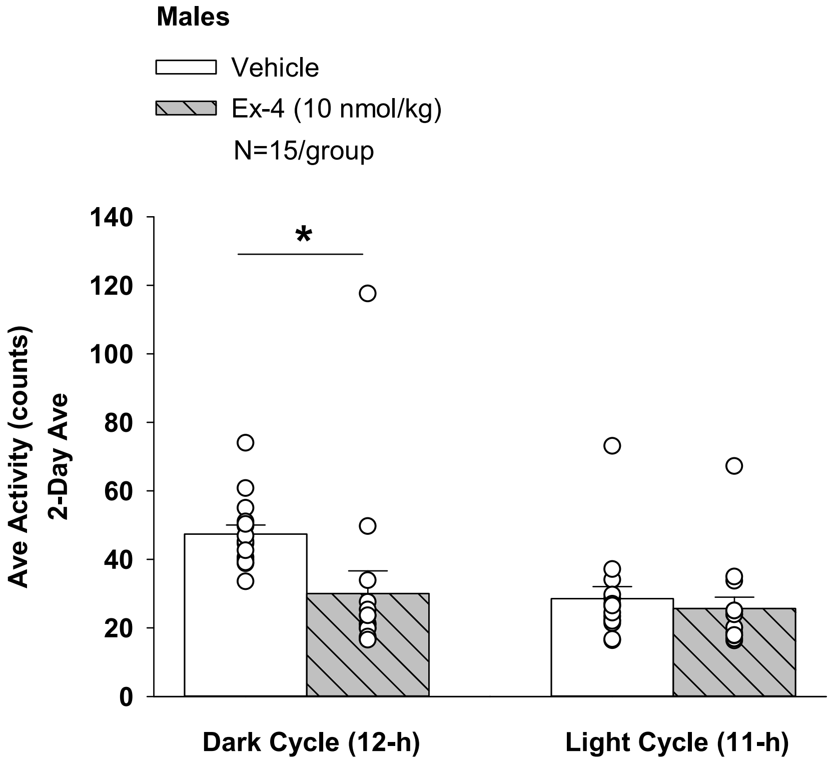

Supplemental Figure 4B

bioRxiv preprint doi: https://doi.org/10.1101/2025.01.06.631534; this version posted February 3, 2025. The copyright holder for this preprint (which was not certified by peer review) is the author/funder. This article is a US Government work. It is not subject to copyright under 17 USC 105 and is also made available for use under a CC0 license.

Supplemental Table 1. Range of detectability for plasma hormones

Plasma hormone	Range of Detectability
Adiponectin	0.25-10 ng/mL
FGF-21	0.0313-2 ng/mL
Glucagon	6.97-633.9 pg/mL
Insulin	0.069-50 ng/mL
Irisin	78-5,000 ng/mL
Leptin	0.07-51.9 ng/mL
